# Thermal treatment enhances the resisting exercise fatigue effect of *Phyllanthus emblica* L.: novel evidence from tannin conversion in vitro, metabolomics, and gut microbiota community analysis

**DOI:** 10.1186/s13020-023-00835-4

**Published:** 2023-10-01

**Authors:** Dingkun Zhang, Xuan Deng, Mengqi Li, Min Qiu, Yifan Zhang, Gefei Li, Yurou Jiang, Peng Tan, Sanhu Fan, Youde Zheng, Junzhi Lin, Li Han, Haozhou Huang

**Affiliations:** 1 State Key Laboratory of Southwestern Chinese Medicine Resources, Pharmacy College, Chengdu University of TCM, Chengdu, 611137 China; 2 Pharmacy Department, Sichuan Nursing Vocational College, Chengdu, 610100 China; 3 State Key Laboratory of Quality Evaluation of Traditional Chinese Medicine, Sichuan Academy of Traditional Chinese Medicine, Chengdu, 610041 China; 4 Sanajon Pharmaceutical Group, Chengdu, 610000 China; 5 TCM Regulating Metabolic Diseases Key Laboratory of Sichuan Province, Hospital of Chengdu University of Traditional Chinese Medicine, Chengdu, 610072 People’s Republic of China; 6 State Key Laboratory of Southwestern Chinese Medicine Resources, Innovative Institute of Chinese Medicine and Pharmacy, Chengdu University of Traditional Chinese Medicine, Chengdu, 611137 China; 7 Meishan Affiliated Hospital of Chengdu University of Traditional Chinese Medicine, Meishan Traditional Chinese Medicine Hospital, Meishan, 620010 China

**Keywords:** *Phyllanthus emblica* L., Ellagic acid, Gallic acid, Drying method, Resisting exercise fatigue effect

## Abstract

Polyphenols are the main component of *Phyllanthus emblica* (PE). However, polyphenols are so easy to transform that it is unknown that how drying methods driven by heating affect the anti-fatigue effect of PE. This manuscript investigated the effects of five drying methods on the chemical composition transformation and anti-fatigue of PE, and discussed the action mechanism. The results suggested that the anti-fatigue effect of PE with hot-air-dried at 100 °C was the best, which was as 1.63 times as that with freeze-drying. Ellagic acid (EA) may be a key component of PE in anti-fatigue, and its mechanism of action may be related to regulating intestinal microbiota, protecting mitochondria, and regulating energy metabolism. This study first revealed the thermal transformation of polyphenols in PE, found the most effective strategy for enhancing the anti-fatigue function, and explores its action mechanism.

**Supplementary Information:**

The online version contains supplementary material available at 10.1186/s13020-023-00835-4.

## Introduction

In 1982, the Fifth International Conference on Exercise Biochemistry proposed fatigue is that a particular intensity of activity during the course of physiological activities can’t be initiated or maintained by the body [19]. Fatigue can generally be categorized into three types, including physiological fatigue, pathological fatigue and psychological fatigue. The peripheral fatigue, one type of physiological fatigue, also named as muscle fatigue and sports fatigue, primarily caused by muscle factors, including energy deficiency, metabolite accumulation and metabolic disorder in skeletal muscle [14, 29, 35]. Although peripheral fatigue is not a clinical symptom, it will have major negative consequences on the body and result in a number of diseases if it is not actively and successfully treated [27]. Under today’s fast-paced living conditions, phenomena like unreasonable diet, lack of exercise, irregular work and rest, lack of sleep, mental tension, high psychological pressure, and long-term bad mood are widespread. As a result, more and more people are in sub-health state (a special state between health and illness) and facing “unexplained fatigue” [26]. At the same time, in recent years, sports fatigue has received an increasing amount of study and attention in the disciplines of athletics, the military, and aerospace. The rapid eradication of fatigue has become a focus of global study. And the dietary supplements or natural anti-fatigue products are gaining popularity.

*Phyllanthus emblica* L. (PE) is a tropical fruit of Euphorbiaceae, which is sweet, sour, crisp and slightly astringent. With a history spanning thousands of years, it is extensively dispersed in tropical and subtropical nations like India, Southwest China, Vietnam, Thailand, and Indonesia. PE is frequently utilized as both a fruit and a medication, and typically used in the form of a decoction and pill powder as a medication. PE was initially introduced to China from Ayurvedic medicine in India, which then developed in Chinese medicine and was widely used. Traditional Chinese medicine conside the use of PE for a prolonged period of time can strengthen qi and lighten the body [5, 20]. At the same time, Triphala powder, which is made up of PE, Terminalia chebula and Terminalia bellirica, is often used to treat fatigue in the early and late stages of plague fever [13, 31]. Additionally, current pharmacological study demonstrates PE's blatant anti-fatigue action. For instance, the swimming time of normal mice can be significantly extend by gavag 0.3 and 0.5 g kg^−1^ of PE water extract for 30 days [6]. Rats undergoing high-intensity endurance training can have their fat decomposition promoted and their glycogen consumption reduced by the administration of 3.5 g kg^−1^ PE with 49 days [22].

Polyphenols are the main active ingredients in PE, including as chebulagic acid, corilagin, gallic acid (GA) and ellagic acid (EA) [4, 10–12]. They have the effects of inhibiting oxidation, maintaining mitochondrial function and energy metabolism, regulating bone, sugar, lipid and protein metabolism, regulating intestinal flora, and protecting nerve, which may be the material basis for the anti-fatigue [15, 32, 38, 39]. The polyphenols in PE, however, are unstable and easily change when heated during processing. Polyphenols primarily experience hydrolysis, oxidation and polymerization that greatly affect their composition and activity. For instance, when tea soup was heated, the higher the temperature is, the longer the heating period, and the quicker the polyphenols are transformed, the more obvious the changes in the appearance, flavor and activity of tea soup will be Kim et al. [16]. Similar phenomena are reported in wine and coffee [7, 30]. The hydrolysis of polyphenols will inevitably affect PE’ s efficacy, but now, there is no report on the composition change law in the drying process of PE and no concern regarding the impact of this process on the anti-fatigue effect, making it challenging to choose a suitable processing method of PE. This is the main cause of the inconsistent and unstable nature of PE products, which prevents the high-quality growth of the PE relevant sectors.

At the same time, the anti-fatigue mechanism of PE is still unclear. Studies have found that the generation of exercise-induced fatigue is closely related to energy metabolism. And polyphenols and some of their metabolic derivatives (like urolithin) have been confirmed to regulate energy metabolism by protecting mitochondria [9, 32] (Zhao 2021; Zhou and Jiang 2019). However, the transformation and absorption of polyphenols in vivo requires the participation of intestinal bacteria. Therefore, the anti-fatigue mechanism of PE may be related to the mitochondria and intestinal flora, which needs further elucidation.

To sum up, in light of the aforementioned issues, this paper explored PE’s chemical composition variations and transformation laws using various drying methods, reserched the impact of various processing methods on PE’s anti-fatigue properties and explored the mechanism of anti-fatigue of PE from the perspective of intestinal flora, metabolism and mitochondria. The goal of this study was to better understand the connection between PE’s anti-fatigue properties and its heat transformation law during the processes. This is significant in terms of investigating the physical basis of PE’s anti-fatigue properties, improving PE’s production method, and creating associated dietary supplements.

## Materials

### Chemicals and reagents

GA, corilagin and EA reference materials purchased from Chengdu Biopurify Phytochemicals Ltd. (batch No. 4051109, PRF7102406 and PRF7101305, with purity ≥ 98%). Chebulagic acid reference materials purchased from Chengdu Chroma-Biotechnology Co., Ltd. (batch No. chb171222, with purity ≥ 98%). Water was ultra pure water, and methanol and phosphoric acid were chromatographic grade manufacturers. Lactate dehydrogenase (LDH), lactic acid (LA), blood urea nitrogen (BUN), creatine kinase (CK), glucose (GLU), hepatic glycogen (HGLY), muscle glycogen (MGLY), glutathione peroxide (GSH-Px), malondialdehyde (MDA), and ATP ase biochemical reagent kit purchased from Nanjing Jiancheng Bioengineering Institute. JC-1 mitochondrial membrane potential test box was purchased from Wuhan Servicebio.

### Sample source and dry fruit preparation of PE

Fresh fruit of PE was purchased from Fujian, China. And all PE samples were divided into 5 parts, which were freeze dried for 239 h (FD), dried in the sun for 185 h (DS), hot-air dried at 40 °C for 75 h, hot-air dried at 70 °C for 22 h and hot-air dried at 100 °C for 8.5 h. The water content of the prepared dry samples is less than 10%.

### Determination of chemical constituents in PE

#### Assay by HPLC

Take 1 mg of GA, chebulagic acid, corilagin, epicatechin and EA reference materials respectively, put them in different measuring flasks, and add 50% methanol (EA is dissolved in methanol) to dissolve and dilute to 10 ml, shook well, and passed 0.22 μM microporous filter membrane, which prepared into 0.1 mg ml^−1^ control solution.

Accurately weigh 0.1 g of FD, DS, 40 °C, 70 °C and 100 °C PE samples, and placed them in conical flasks. Then, accurately added 10 ml of 50% methanol water, weigh the mass, ultrasonic for 20 min (300 W, 40 kHz), cooled them, added 50% methanol to make up the lost mass, shook them well, and passed 0.22 μM microporous filter membrane.

The column, a Welchrom C18 column (4.6 mm × 250 mm, 5 μm), was maintained at 30 °C. Solvents used for separation were methanol (eluent A) and 0.05% phosphoric acid in water (v/v) (eluent B). The gradient used was: 0–6 min, maintain at 5% A. 6–15 min, linear gradient from 5 to 7% A. 15–20 min, linear gradient from 7 to 15% A. 20–25 min, linear gradient from 15 to 21% A. 25–31 min, linear gradient from 21 to 22% A, maintain at 22% A until 41 min. 41–47 min, linear gradient from 22 to 28% A. 47–51 min, linear gradient from 28 to 32% A. 51–57 min, linear gradient from 32 to 38% A. 57–70 min, linear gradient from 38 to 45% A. 70–80 min, linear gradient from 45 to 65% A. 80–85 min, linear gradient from 65 to 5% A [24]. The flow rate was 1.0 ml min^−1^. Detection wavelength was 270 nm. The sample injection volume was 10 μl.

#### Data analysis

The HPLC data of PE were analyzed by using the similarity evaluation system of Chromatographic Fingerprint of Traditional Chinese Medicine (2012 a) to calculate the similarity of fingerprint. Cluster heat map analysis and principal component analysis (PCA) were carried out by using xcms package of R. SIMCA 13.0 statistical analysis software was used for S-plot analysis.

### Fatigue resistance evaluation

#### Preparation of PE extract

According to the results of chemical analysis, the FD, 40 °C and 100 °C with the largest difference in the content of main active ingredients were selected for further anti fatigue efficacy verification. Take the FD, 40 °C, 100 °C powder, add ten times the amount of water, extract by ultrasonic for 20 min, filter with gauze to remove the dregs, prepare 0.1 g ml^−1^ solution, and store it in the refrigerator at − 20 °C.

#### Animal and experimental design

50 male SPF mice at eight weeks of age and weighting 18–22 g were obtained from the Chengdu Da Shuo Biotechnology Co., Ltd., Chengdu, China. The mice were randomly divided into blank static group, fatigue model group, FD group, 40 °C group, 100 °C group, with 10 mice in each group. The dose of 1.5 g kg^−1^ d^−1^ was administered continuously for 18 days. The mice were killed by intraperitoneal injection of Pentobarbital sodium. This experiment met the requirements of animal experimental ethics and was approved by the Experimental Animal Ethics Committee of Chengdu University of Traditional Chinese Medicine (approval number: 2020-28).

The grasping force of mice’s forelimbs was measured on the 13th day of administration. The treadmill running exhaustion test (20 m/min, 0–20 min; 25 m/min, 21–25 min; 30 m/min, 26–30 min) was carried out 30 min after administration on the 14th day. The rod rotation exhaustion test (30 r/min, 0–20 min; 40 r/min, 21–25 min; 50 r/min, 26–30 min) was carried out 30 min after administration on the 16th day, On the 18th day, 30 min after administration, the mice were subjected to weight bearing exhaustive swimming test (10% weight), and the time of exercise exhaustion was recorded.

#### Collection of serum and gastrocnemius samples

30 min after the end of swimming test, blood was taken and centrifuged (4 °C, 3500 r/min, 15 min) to obtain serum. The liver and gastrocnemius of mice were excised and stored in the refrigerator at − 80 °C with the serum.

#### Determination of biochemical indexes

Take mouse serum, and measure the content of LDH, LA, BUN, CK and GLU in serum according to the instructions of the kit. Take the mouse liver, and measure HGLY content according to the instructions of the kit. Take the gastrocnemius muscle of mice and measure the content of MGLY, GSH-Px, MDA, and Na^+^–K^+^–ATP ase in gastrocnemius muscle according to the instructions of the kit.

### Spectrum effect correlation analysis

The correlation analysis was carried out between the differential compounds measured in the drying stage of PE and the related evaluation indexes of anti-fatigue. Among that, the differential compounds of PE were determined as independent variables, and the anti-fatigue indexes were determined as dependent variables.

### Exploration of key components and mechanisms of action in anti fatigue

#### Preparation of liquid medicine

Take an appropriate amount of PE and extract it to prepare a 0.15 g ml^−1^ medicinal solution. Take an appropriate amount of GA and EA, and add RO water to prepare a reference solution of 3.4 mg ml^−1^ and 1.41 mg ml^−1^, respectively.

#### Animal and experimental design and pharmacodynamic evaluation

Divide 50 mice into control group, fatigue model group, PE group, GA group, and EA group, with 10 mice in each group. The content of GA and EA in PE dried at 100 °C was 2.3% and 0.94%, respectively. Therefore, PE, GA, and EA were continuously administered at doses of 1.5 g kg^−1^ d^−1^, 34 mg kg^−1^ d^−1^, and 14.1 mg kg^−1^ d^−1^, respectively, for 18 days. Determine phenotypic and biochemical indicators under items 2.4.2–2.4.4.

#### Determination of gut microbiota

After swimming experiment, the feces of each group of mice were collected uniformly into sterile tubes in a sterile environment. Each group was randomly divided into 7 parallel tubes and stored at − 80 °C. Total DNA was extracted using Stool DNA Kit, amplified by PCR based on 16S rDNA gene, and sequenced using Illumina MiSeq platform. Using Omicrostudio software to calculate Observed otus, Chao1, Shannon, Simpson, etc.

#### Determination of serum metabolomics

##### Collection and pre-treatment of serum samples

Take serum sample (200 μl) Add pre cooled methanol (600 μl) Mix evenly, vortex for 60 s, let stand in a refrigerator at 4 °C for 30 min, centrifuge at 13,000 r·min^−1^ for 10 min, collect the supernatant, and evaporate and dry under a gentle nitrogen flow. Using methanol (200 μl) dissolve the sample again, vortex oscillate for 60 s, centrifuge at 13,000 r min^−1^ at 4 °C for 10 min, and take the supernatant for backup.

##### Chromatographic conditions

Mobile phase A is a 0.1% formic acid aqueous solution and mobile phase B is an acetonitrile solution. Gradient elution time is 0–3 min, 2%–2% B; 3–5 min, 2%–7%B; 5–15 min, 7%–21%B; 15–20 min, 21%–78%B; 20–21 min, 78%–85%B; 21–24 min, 85%–95%B; 24–26 min, 95%–95%B; 26–28 min, 95%–2%B; 28–30 min, 2%–2%. The column temperature is 40 °C, the flow rate is 0.3 ml min^−1^, and the injection volume is 3 μl.

##### Mass spectrometry conditions

The positive and negative ion modes of the electric spray ion source (ESI) are used for detection and analysis. The capillary voltage is 4 kV, the cone voltage is 50 V, and the ion source temperature is 150 °C. The atomized gas is high-purity nitrogen, with a conical gas flow rate of 50 l h^−1^, a desolvent gas flow rate of 600 l h^−1^, and a temperature of 250 °C. Mass spectrometry data was collected in an ion scanning range of m/z50-1200. Use leucine enkephalin (LE) for calibration during data collection. In the negative ion mode, the exact relative molecular weight of LE [MH]—is calculated as M/z 554.2615.

##### Data processing and multivariate analysis

The QI software is used to extract the features of the original data, and the data matrix file containing the information of metabolite retention time (RT), mass-to-charge ratio, intensity, etc. is obtained. Import it into SIMCA-P 14.0 software for principal component analysis. Perform log transformation on the original data, perform orthogonal partial least squares discriminant analysis, and draw an s-plot plot. Variables with variable importance in projection (VIP) > 3.5 and | p (corr) |> 0.58 are considered differential variables.

##### Metabolic pathway and functional analysis

Import the screened differential compounds into MetaboAnalyst 5.0 (http://www.metaboanalyst.ca/) to conduct path analysis through KEGG values. Metabolic pathways with an impact value greater than 0.01 are considered potential target pathways.

#### Pathological observation, muscle fiber and mitochondrial ultrastructure observation of gastrocnemius muscle

Some of tissue of gastrocnemius muscle were fixed in 10% paraformaldehyde, dehydrated, transparent, dipped in wax and embedded. After staining with eosin dye, the morphological changes of skeletal muscle tissue were observed with light microscope. The other tissue was fixed with 2.5% glutaraldehyde buffer and ultrathin sections were made. The ultrastructure of muscle fibers and mitochondria were observed under transmission electron microscopy.

#### Detection of mitochondrial membrane potential

Take fresh liver tissue, add nine times the amount of physiological saline, and perform low-temperature homogenization. Liver tissue homogenate was centrifuged at 2000 r min^−1^ for 10 min at 4 °C. Take the supernatant and centrifuge at 10,000 r min^−1^ for 15 min at 4 °C. Remove the supernatant (cytoplasm) and prepare liver mitochondria. The membrane potential was measured with JC-1 mitochondrial membrane potential detection kit.

## Results and discussion

### Effects of different processing methods on chemical composition

#### Chromatographic analysis of PE samples

**Fig. 1:**
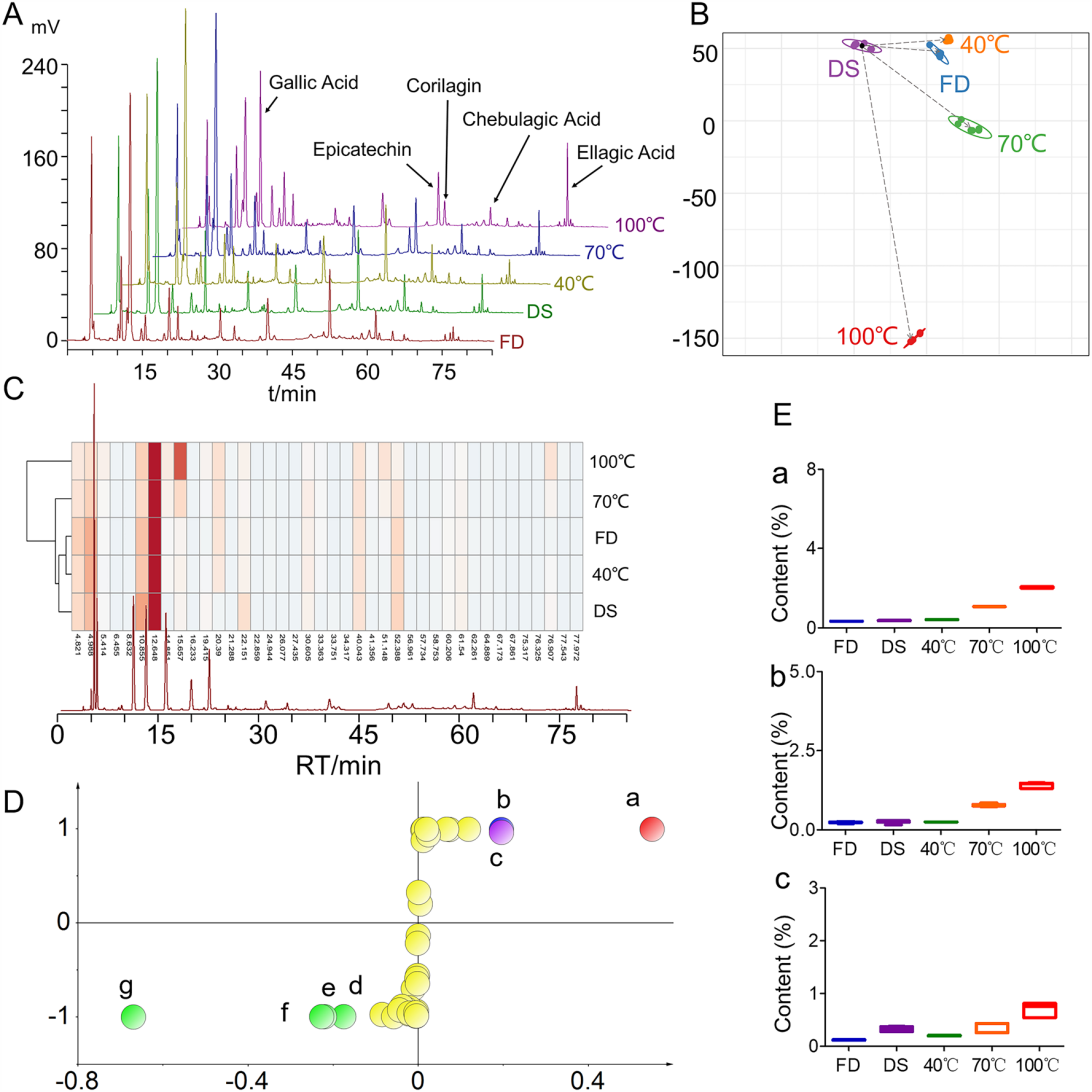
Chromatogram (**A**), PCA score chart (**B**), heat map (**C**), S-plot results (**D**) and content comparison (**E**). a. gallic acid, b. corrilagin, c. ellagic acid, d. Compound 1 (4.82 min), e. Compound 2 (4.99 min), f. Compound 3 (52.40 min), g. Compound 4 (12.65 min)

Analyze the chromatograms of the test samples of PE prepared by different drying methods, and identify the relevant components, as shown in Fig. 1A. There is a significant difference in the total peak area of PE during the drying stage, with the difference between PE dried at 100 °C and other dried fruits being the largest. This indicates that the temperature during the drying stage has a significant impact on the composition and content of PE.

#### Systematic cluster analysis and principal component analysis

Using the peak area of common peaks in PE as a variable, PCA analysis and cluster heat map analysis were performed on five dried samples of PE, as shown in Fig. 1B and C. After drying, there were significant differences in the components of PE, mainly appearing at peaks with retention times of 12 min, 15 min (GA), and 76 min (EA). The sample clustering can be divided into two categories, among which low-temperature dried samples (FD, DS, 40 °C, 70 °C) are clustered into one category, and high-temperature dried (100 °C) is clustered into one category. The different drying methods will have a significant impact on the composition and content of the components of PE, especially high-temperature dried PE and low-temperature dried PE. This indicates that heating may be the main reason for the changes in the composition of PE during the drying stage.

#### Comparison of main active components in PE

Use S-Plot analysis to identify the differential chemical markers between different dried products of PE, as shown in Fig. 1D. There are 7 significantly different compounds in different dried products of PE, among which 3 components have been identified as GA, EA, and corilagin. Subsequently, we compared their contents, as shown in Fig. 1E. There is a significant positive correlation between the content of GA, EA, and corilagin and drying temperature. The order of content is: 100 °C > 70 °C > 40 °C > DS > FD. The higher the drying temperature, the higher the content of GA, EA, and corilagin. Heating can promote the hydrolysis of large molecule polyphenols and the production of small molecule polyphenols (GA, EA).

### Effects of different processing methods on anti fatigue efficacy

#### Phenotypic indicators in fatigue resistance evaluation

**Fig. 2:**
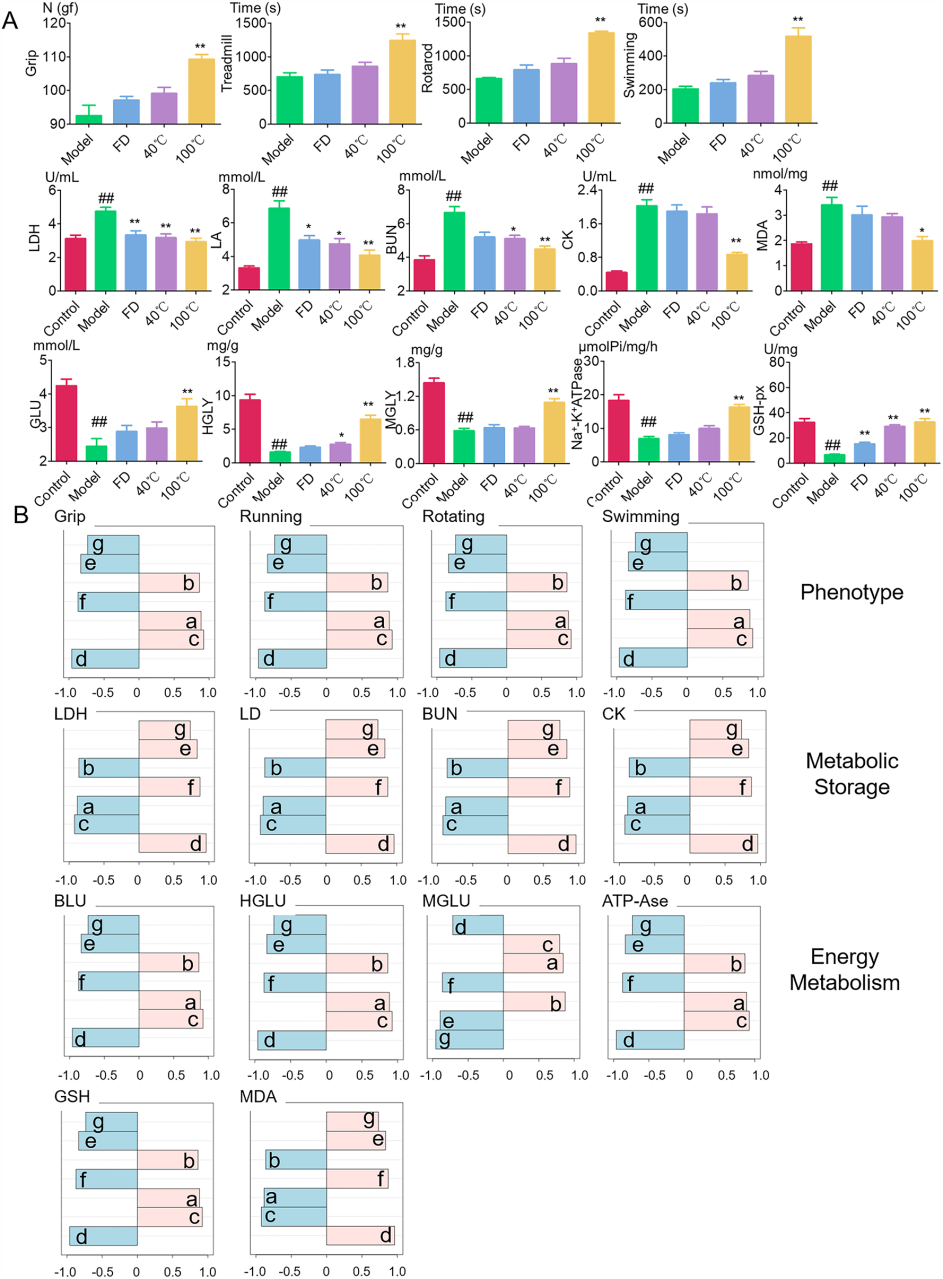
Determination results of anti-fatigue related indicators (**A**) and correlation analysis of different compounds and anti-fatigue effects during the processing of PE (**B**). a. gallic acid, b. corrilagin, c. ellagic acid, d. Compound 1 (4.82 min), e. Compound 2 (4.99 min), f. Compound 3 (52.40 min), g. Compound 4 (12.65 min)

The results of grip strength and exhaustion time of treadmill, rotarod, and swimming are shown in Fig. 2. The results showed that the mice’s grip increased and the exercise time (treadmill, rotarod and swimming) prolonged after administration. There was a significant difference in grip and exercise time between 100 °C group and model group (p < 0.05). For the ability of PE to enhance grip and improve exercise endurance, different drying methods will show significant differences, mainly manifested as 100 °C > 40 °C > FD. The higher the temperature during drying, the stronger the ability of PE to alleviate muscle fatigue.

#### Reduce the accumulation of metabolites

LA and BUN are products of glycolysis and amino acid metabolism, while LDH and CK are key indicators reflecting fatigue and evaluating muscle cell damage. This study measured the levels of LA, LDH, BUN, and CK, as shown in Fig. 2A. Compared with the control group, the model group showed a significant increase in LA, LDH, BUN, and CK levels (p < 0.01), indicating that excessive exercise increased the levels of LA, LDH, BUN, and CK in mice, leading to a large accumulation of metabolites in the body. After administration of PE, the levels of LA and LDH significantly decreased (FD, 40 °C group LA, p < 0.05; other groups, p < 0.01), BUN and CK levels significantly decreased (p < 0.01) in the 100 °C group, and BUN levels significantly decreased (p < 0.05) in the 40 °C group. This indicates that PE can reduce the accumulation of metabolic substances, improve muscle damage, exert anti exercise fatigue effects, and prolong the exercise time of mice. At the same time, different drying methods will cause differences in the ability of PE to reduce the accumulation of metabolites and alleviate muscle fatigue, with the ability ranking as 100 °C > 40 °C > FD. This indicates that the higher the temperature during the drying process, the stronger the ability of PE to reduce the accumulation of metabolic products and alleviate muscle fatigue.

#### Regulate oxidative stress

Under normal circumstances, the production of ROS in the body and the antioxidant system are in dynamic equilibrium. However, excessive ROS generated by vigorous exercise can lead to imbalanced oxidative stress reactions and the formation of lipid peroxidation product MDA, which can alter the fluidity and permeability of cell membranes, thereby causing damage to cell structure and function. GSH-Px is an endogenous antioxidant enzyme that plays an important role in maintaining the balance of antioxidant stress in the body [27]. This study measured the levels of GSH-Px and MDA. The results in Fig. 2A showed that compared to control group, the GSH-Px enzyme activity in model group was significantly reduced (p < 0.01), while the MDA content was significantly increased (p < 0.01), indicating that vigorous exercise induced oxidative damage in the mouse body. After administration of PE, the levels of GSH-Px significantly increased (p < 0.01), while the MDA content in the 100 °C group significantly decreased (p < 0.05). It indicates that PE can exert its anti-exercise fatigue effect by reducing oxidative damage in the body and prolonging the exercise time of mice. The order of antioxidant capacity of PE prepared by different drying methods is 100 °C > 40 °C > FD. The higher the drying temperature, the stronger the antioxidant capacity of PE.

#### Regulate energy metabolism

Increase energy reserves: GLY and GLU are the main energy substances in the body, and the consumption of energy reserves can also lead to exercise fatigue. This study measured GLY and GLU levels in mice, as shown in Fig. 2A. Compared with the control group, the HGLY, MGLY and GLU levels in model group were significantly reduced (p < 0.01). Excessive exercise consumed a large amount of energy substances in the mouse body. After administration of PE, 100 °C PE significantly increased the content of HGLY, MGLY, and BLU (p < 0.01), while 40 °C PE significantly increased the content of HGLY (p < 0.05). This indicates that PE can exert its anti-exercise fatigue effect by increasing the body’s energy reserve.

Maintaining mitochondrial potential balance: Excessive exercise can cause metabolic disorders of Ca^2+^, K^+^, and Na^+^ ions, resulting in potential imbalance and mitochondrial damage, thereby affecting ATP production and energy metabolism. This study measured the content of Na^+^–K^+^–ATPase. The results are shown in Fig. 2A. Compared with the control group, the Na^+^–K^+^–ATPase activity of the model group was significantly reduced (p < 0.01). Severe exercise disrupted the balance of mitochondrial potential in the body and hindered ATP synthesis. After administration of PE, the activity of Na^+^–K^+^–ATPase increased, and there was a significant difference (p < 0.01) between the 100 °C group and the model group, indicating that PE can exert anti exercise fatigue effects by maintaining normal mitochondrial function.

Meanwhile, there are significant differences in the ability of PE prepared by different drying methods to regulate energy metabolism, with the order being 100 °C > 40 °C > FD. The higher the drying temperature, the stronger the ability of PE to increase energy reserves, balance mitochondrial potential, and promote energy production.

#### Spectral effect correlation analysis

The correlation analysis was conducted between the differential compounds measured in the drying stage of PE and the resisting exercise fatigue related evaluation indicators, as shown in Fig. 2B. The results showed that the components positively correlated with the resisting exercise fatigue ability were GA, EA, and corilagin, while compounds 1–4 showed a negative correlation. Among them, in terms of enhancing grip, prolonging exercise time, inhibiting harmful metabolites (CK, LA, LDH, BUN), enhancing energy metabolism (ATP, GLU, HGLY), enhancing GSH-Px enzyme activity, and reducing MDA content, the order of dominant differential compounds is: EA > GA > corilagine. In terms of enhancing the ability of MGLY, the correlation order of different compounds is as follows: Corilagin > GA > EA.

### Antifatigue evaluation of EA and GA

#### Phenotypic indicators

**Fig. 3:**
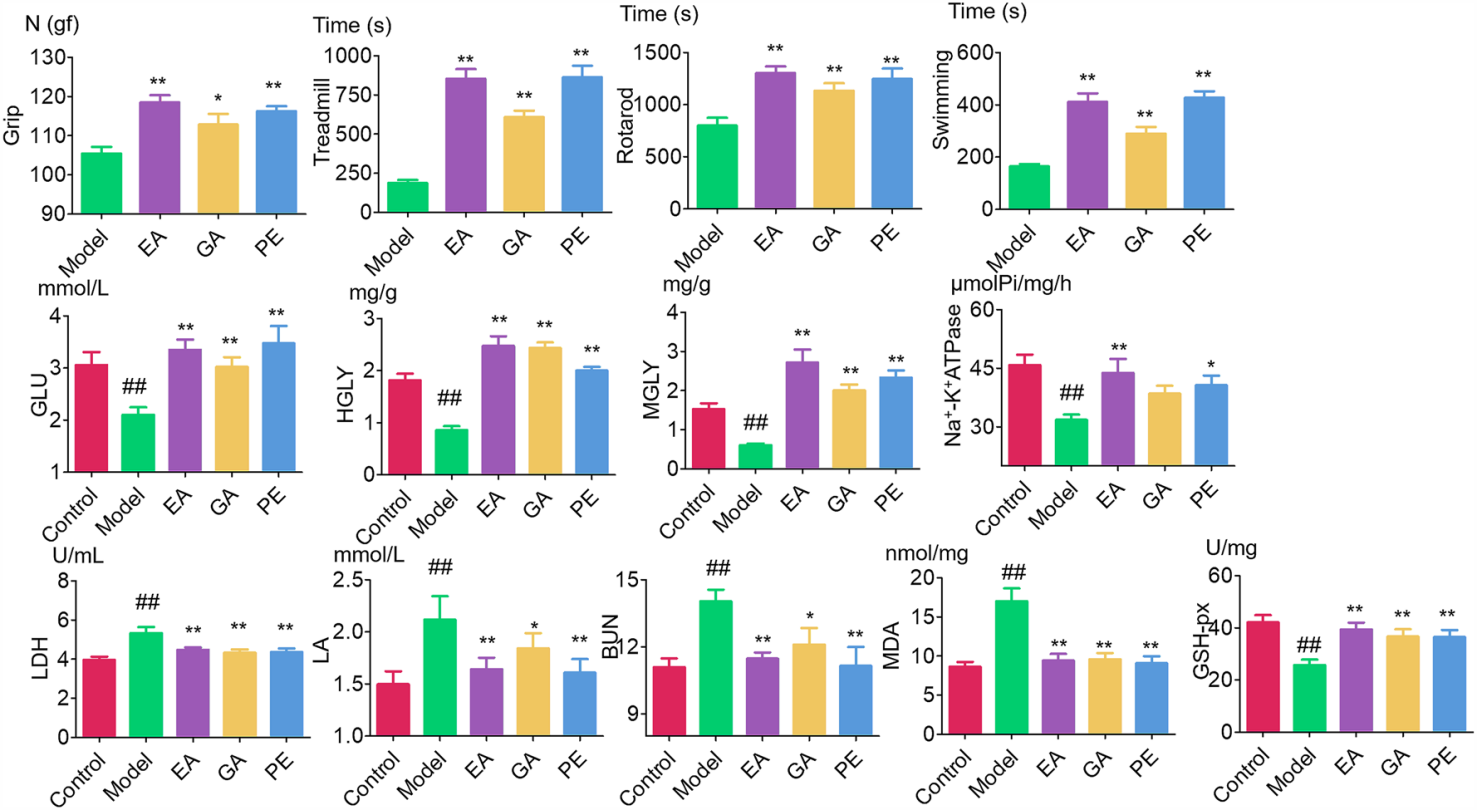
Measurement results of antifatigue related indicators of PE, EA, and GA

The results of the exhaustion experiments on grip, running platform, rotating rod, and swimming are shown in Fig. 3. The results show that the treadmill, rotarod, and swimming time in PE, GA, and EA groups is significantly prolonged, which has extremely significant differences compared to the model group (p < 0.01). The grip of mice significantly increased, with significant differences (PE and EA group, p < 0.01; GA group, p < 0.05). This indicates that EA and GA can prolong the exercise time. GA and EA may be active ingredients of PE to improve exercise endurance in mice, and EA has stronger anti-fatigue activity than GA.

#### Biochemical indicators

The measurement results of energy metabolism, oxidative stress, and harmful metabolite accumulation related indicators are shown in Fig. 3. The measurement results of energy metabolism indicators: after administration, the GLU, ATPase, HGLY, and MGLY levels in the PE, GA, and EA groups were significantly increased. Except for the level of GA group’s ATPase, which was not significantly different from the model group, other indicators in the other groups showed extremely significant differences from the model group (p < 0.01). The measurement results of oxidative stress related indicators showed that compared to the control group, the GSH-Px levels in the model group mice were significantly reduced (p < 0.01), while the MDA levels were significantly increased (p < 0.01). After administration, the levels of GSH-Px in PE, GA, and EA group significantly increased (p < 0.01), while MDA significantly decreased (p < 0.01). The measurement results of harmful metabolite related indicators showed that compared to the control group, the levels of LDH, LA, and BUN in the model group mice were significantly increased (p < 0.01). After administration, the levels of LDH, LA, and BUN were significantly reduced in the groups of PE, GA, and EA (p < 0.05). This indicates that EA and GA can reduce the accumulation of harmful metabolites, regulate oxidative stress, and regulate energy metabolism, and the effect of EA is stronger than that of GA.

### Analysis of gut microbiota results

#### Analysis of common species

**Fig. 4:**
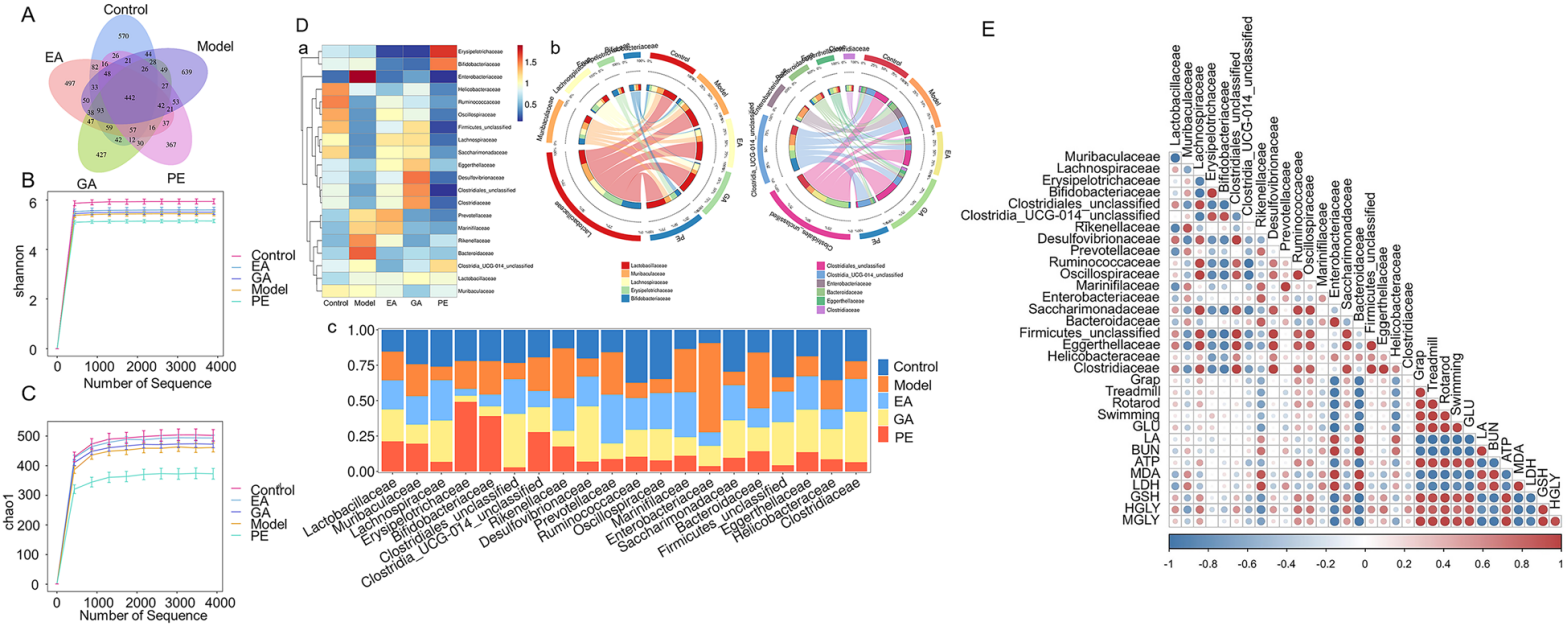
Metabolomics results. **A** Venn chart; **B** Shannon dilution curve, **C** Chao1 Index chart; **D** correlation analysis of intestinal flora abundance (family level): a: Heat map; b: Circos (the left part is the abundance information of the family with large abundance changes and its corresponding abundance information, and the right part is the grouping information); c: Histogram of intestinal flora abundance in each group; **E** correlation between Gut microbiota and anti-fatigue effect

Take 7 fecal samples from each group for sequencing. A total of 2,272,664 high-quality sequences were generated. Using QIIME2 software to process the original sequencing data, a total of 3939 OTUs were obtained through homology comparison and cluster analysis. Draw a Venn diagram based on the classification results of each group of OTUs. As shown in Fig. 4A, the number of unique OTUs in the control group is 570, the model group is 639, the PE group is 367, the tannic acid group is 497, and the GA group is 427 OUT. After administration, there were fewer unique OTUs in mice feces.

#### α Diversity analysis

The dilution curve is a curve constructed based on the amount of sequencing data and species diversity. The smoothness of the curve reflects the impact of sequencing depth on the diversity of observed samples. The Shannon dilution curve of the five samples tends to flatten, as shown in Fig. 4B, indicating that the number of extracted samples meets the standard, and the depth of sequencing data basically includes sufficient sample types. The Chao1 index is used to reflect the richness of the bacterial community in the sample. The richness results of Chao1 bacterial community are shown in Fig. 4C. Compared with the blank group, the Chao1 index of the model group decreased. Compared with the model group, the Chao1 index of the EA and GA groups slightly increased, while the PE group significantly decreased. It indicates that PE may have the effect of regulating the transformation of intestinal microbiota to specific microbiota.

#### Changes in microbial abundance at different classification levels

At the family level, the top 20 species of bacteria in the feces of mice in each group include Lactobacillus, Lachnospiraceae, Erysipelotrichaceae, Enterobacteriaceae, Clostridiales, Bacteroidaceae, Bifidobacteriaceae, Eggerthellaceae, etc. See Fig. 4D, where Enterobacteriaceae is related to the production of lactic acid [25]. Lactobacillus is closely related to the production of the key inhibitory neurotransmitter 5-HT in the regulation of the central nervous system [1, 21, 23, 28, 36]. Bifidobacteriaceae participates in regulating body metabolism [25]. Lactobacillus, Bacteroidaceae, Clostridiales, Bifidobacteriaceae and Eggerthellaceae are involved in the metabolic transformation of EA [3, 8, 18, 33, 34, 37]. Compared with the control group, the proportion of Lachnospiraceae and Clostridiales in the model group decreased, while the proportion of Erysipelotrichaceae and Enterobacteriaceae increased significantly. After administration, the Enterobacteriaceae in the PE, GA and EA groups significantly decreased, the proportion of Spirillaceae and Clostridaceae in the EA and GA groups increased, and the Erysipelotrichaceae significantly decreased, close to the control group, indicating that the PE, EA and GA can, to a certain extent, reduce the impact of excessive fatigue on intestinal microbes, indirectly affect the production of lactic acid, and regulate central nervous system and body metabolism. In addition, for those bacterial communities that have been reported to be involved in the transformation and metabolism of EA, EA increased the proportion of Clostridiales and Eggerthellaceae, GA increased the proportion of Clostridiales, Eggerthellaceae, and Lactobacillus, while PE increased the proportion of Bifidobacteriaceae and Lactobacillus. This indicates that PE, EA, and GA can also promote the conversion and absorption of EA by regulating the composition of gut microbiota, enhancing the effectiveness of the drug. However, the specific types of gut microbiota regulated by the three are different. At the genus level, the bacteria with large changes correspond to the bacteria at the family level, and the results are similar, see Additional file 1: S-1. After administration, Escherichia-Shigella in mice feces were significantly reduced, while Lachnospiraceae and Clostridiales significantly increased, close to the control group. EA and GA also increased the levels of Enterorhabdus genus in the family Eggerthellaceae, while PE significantly increased the levels of Bifidobacterium. However, it is worth mentioning that PE can increase the level of Dubosiella, which has been found in recent studies to improve nematode muscle capacity, delay aging, and extend lifespan. It may be a type of intestinal microbiota that can alleviate fatigue.

Through the Hiplot Pro website (https://hiplot.com.cn/). The correlation between Gut microbiota and anti-fatigue effect was analyzed. The results are shown in Fig. 4E. It is not difficult to see that the microbiota positively correlated with the anti-fatigue effect includes Lactobacillaceae, Muribaculaceae, Lachnospiraceae, Erysipelotrichaceae, Bifidobacteriaceae, Clostridiales_unclassified, Clostridia_UCG-014_unclassified, Rikenellaceae, Desulfovibrionaceae, Prevotellaceae, Ruminococcaceae, Oscillospiraceae, Marinifilaceae, Enterobacteriaceae, Saccharimonadaceae, Bacteroidaceae, Firmicutes_unclassified, Eggerthellaceae, Helicobacteraceae, Clostridiaceae. Among them, the ones with strong correlation are Eggerthellaceae, Lachnospiraceae, Saccharimonadaceae, Ruminococcaceae and Oscillospiraceae. However, among the bacterial communities negatively correlated with anti-fatigue effects, the ones with strongest negative correlation are Enterobacteriaceae and Bacteroidaceae. In summary, the anti-fatigue effect of PE may be related to the regulation of Eggerthellaceae, Enterobacteriaceae etc.

### Serum metabolomics

#### Sample quality control evaluation

**Fig. 5:**
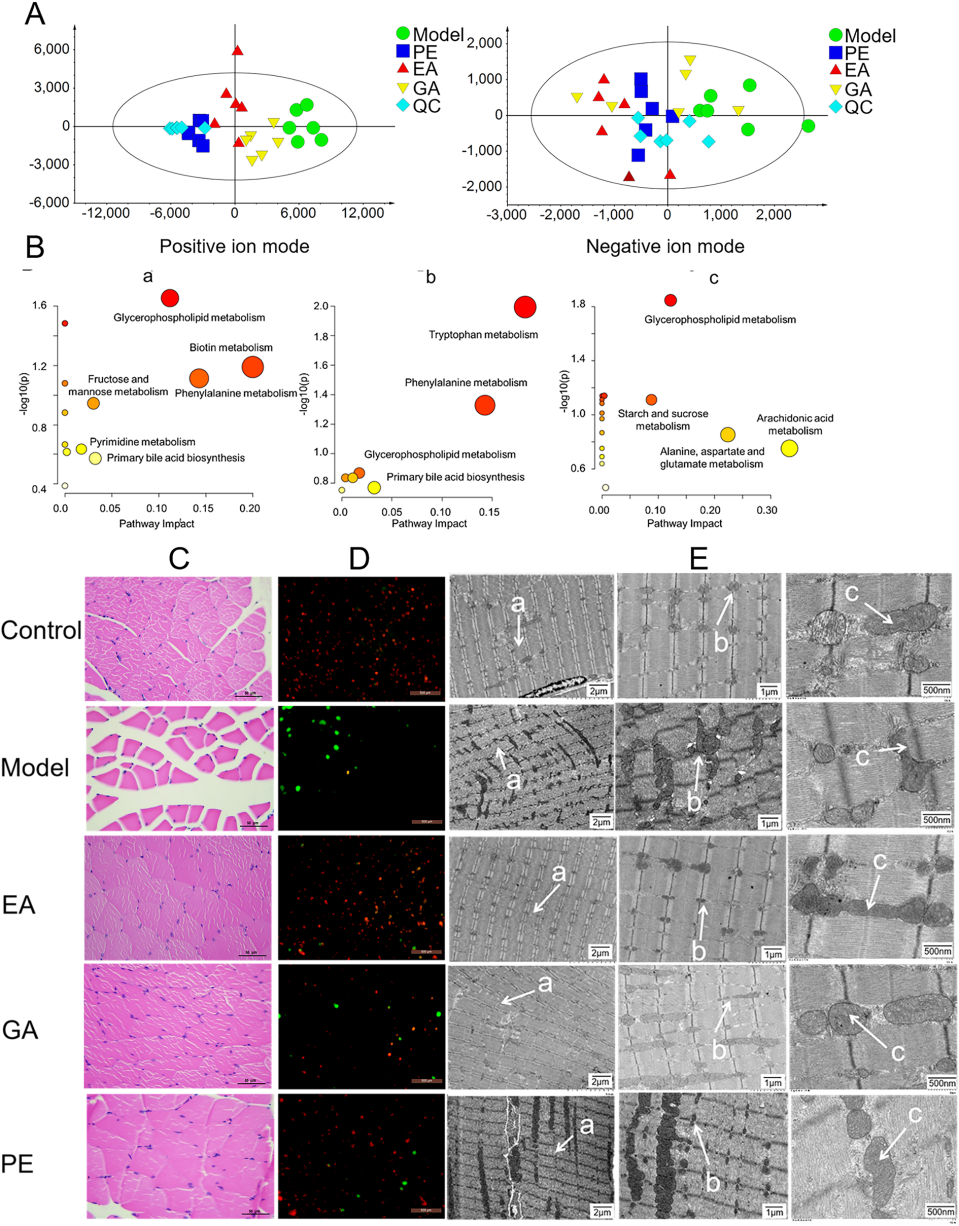
Metabolomics results and observation diagram of skeletal muscle fiber, mitochondrial structure and membrane potential. **A** Positive and negative ion PCA score diagram; **B** enrichment diagram of KEGG pathway of metabolites of PE, EA, and GA; **C** microscopic observation of skeletal muscle HE staining; **D** JC-1 Fluorescence staining of mitochondria; **E** Transmission microscopy of skeletal muscle mitochondria; a: Z-line; b: Mitochondria; c: Cristae

Use unsupervised PCA analysis to evaluate the differences in fecal metabolites between the model group, control group, and administration group. As shown in Fig. 5A, under both positive and negative ion scanning modes, all samples exhibit significant clustering. The obvious aggregation of QC samples indicates that the detection instrument and method are stable, reliable, and have good repeatability, which can further analyze the collected sample metabolomics data. In addition, the groups treated with PE, EA, and GA were well separated from the model group, indicating to some extent that the metabolic profile of mouse serum changed after administration.

#### Analysis of serum differential metabolites between the groups of PE, EA, and GA and the fatigue model group

The use of multivariate statistical analysis (OPLS-DA) was used to determine the changes in metabolites caused by administration, as shown in Additional file 1: S-2–4. From the OPLS-da score chart, it can be seen that there are significant differences in serum metabolites in mice before and after administration. Metabolites with VIP > 3.5 (PE, GA), VIP > 3 (EA), |p (corr)|> 0.58 in the S-plot plot under positive ion mode, and VIP > 3, |p (corr)|> 0.58 in the S-plot under negative ion mode were used as differential metabolites. The final analysis identified differential metabolites between the model group and the PE administration group. Compared with the model group, PE downregulated 6 metabolites, including Clupanodonyl carnitine, Octadecanone, etc., and upregulated 34 metabolites, including Pelargonidin 3-sophoroside 5-glucose, Protocatechuic acid, etc.; There was 1 downregulated (LysoPC) and 37 upregulated (d-Glucosaminide, l-Tryptophan, etc.) of EA; There are 20 downregulated compounds of GA, including Vignic acid B, Mycophenolic acid, etc., and 21 upregulated compounds, including Arginyliisoleucine, 3-*O*-beta-d-glucosyl-brasicasterol. Please refer to Additional file 1: S-5–10 for specific differential compounds.

#### Pathway enrichment analysis

Conduct pathway enrichment analysis on the differential metabolites. The enrichment results are shown in Fig. 5B. The results show that the related pathways of PE, EA, and GA against exercise induced fatigue include 2 glucose metabolism related pathways, 7 lipid metabolism related pathways, 10 amino acid metabolism related pathways, 3 vitamin metabolism related pathways, arachidonic acid metabolism, pyrimidine metabolism, primary bile acid biosynthesis, etc.

Select the KEGG pathway with a concentrated impact value greater than 0.01 as the most relevant pathway. The resisting exercise fatigue effects of PE, EA, and GA are all related to the Glycerophospholipid metabolism pathway. In addition, PE also has significant effects on biotin metabolism, phenylalanine metabolism, primary bile acid biosynthesis, fructose and mannose metabolism, and pyrimidine metabolism. EA also affects tryptophan metabolism, phenylalanine metabolism, and primary bile acid biosynthesis, while GA affects alanine, aspartate and glutamic acid metabolism, starch and sucrose metabolism, and arachidonic acid metabolism. In summary, the metabolic pathways mainly affected by PE, EA, and GA are sugar, lipid, and amino acid metabolic pathways, all of which are related to energy metabolism. In addition, the number of metabolic pathways affected by GA is more abundant than that of EA, but the anti-exercise fatigue effect of GA is not as good as that of EA. This suggests that GA may indirectly assist EA in exerting its anti-exercise fatigue effect by regulating metabolic pathways.

### Effects on muscle fibers and mitochondria

According to the results of metabolomics, the anti-fatigue mechanism of PE may be to act on mitochondria to regulate energy metabolism. To verify this guess, the experiment further observed the effect of PE on mitochondria in fatigue mice.

#### Muscular histological observations

Skeletal muscle fibers are closely related to exercise, and excessive exercise can lead to prolonged muscle tension, leading to pathological changes in muscle fibers. Pathological examination was conducted on the skeletal muscles of mice in the experiment, and the results are shown in Fig. 5C. The muscle fibers and structures of the control group mice are normal, arranged tightly, and there are no inflammatory abnormalities. The muscle fibers of the model group mice contract, decrease in volume, expand in distance, and have obvious gaps. After administration, the muscle gap in mice significantly decreased or even disappeared, indicating that PE, EA, and GA can significantly improve muscle fiber contraction caused by excessive fatigue.

#### Observation of mitochondrial membrane potential

Mitochondrial membrane potential is an important indicator to judge the normal function and structure of mitochondria. In order to explore the protective effect of PE, EA and GA on the mitochondria of mice, the experiment detected the mouse mitochondrial membrane potential, as shown in Fig. 5D. The control group mice mainly showed red fluorescence, indicating that the mitochondrial membrane potential was normal. The green fluorescence in the model group increased significantly, indicating that the mitochondrial membrane potential decreased, and excessive fatigue would lead to the abnormality of the mouse mitochondrial membrane potential. However, after administration of EA, GA and PE, the green fluorescence decreased significantly and the red fluorescence increased significantly, indicating that EA, GA and PE can maintain the normal mitochondrial membrane potential, and protect the mitochondria of fatigue mice to alleviate the damage caused by fatigue.

#### Ultrastructural observation of muscle fibers and mitochondria

The ultrastructure of muscle fibers and mitochondria in mouse skeletal muscles was observed using a transmission microscope, as shown in Fig. 5E. The mice Z line After administration is smooth, and mitochondria are evenly distributed on both sides of the Z line, mostly in a long or oval shape, with clear ridge structures; In the model group, the Z line was irregular, the mitochondria were unevenly distributed, irregular in shape, and different in size. They began to swell, the ridge structure was not clear, and even there were missing and vacuoles. The number of abnormal mitochondria increased, and the electron density decreased. This indicates that PE can effectively improve the mitochondrial structure abnormalities of muscle fibers caused by fatigue.

## Conclusions

One of the key factors influencing variances in food's look, color, chemical makeup, and function is the processing method used, as chemical changes such as oxidation, thermal degradation and thermolysis of main components often occur during processing [2]. Tannin hydrolysis explains why the composition and content of the PE components are dramatically altered after drying. PE, in contrast to other fruits, is high in tannin and contains the galloyl structure and hexahydroxybiphenyl dicarbonyl structure [11]. Following hydrolysis of the ester link created by these two structures and the glucose molecules, GA and EA are produced, respectively. This hydrolysis can happen easily, as long as it is heated or in an enzyme condition. As a result, the dried 100 °C has the maximum level of GA and EA after drying. The hydrolysis of chebulagic acid in PE produces 1 molecule of corilagin and 1 molecule of chebulic acid because the high temperature encourages the hydrolysis of tannin. Colilagin will then hydrolyze to GA and EA [11].

**Fig. 6:**
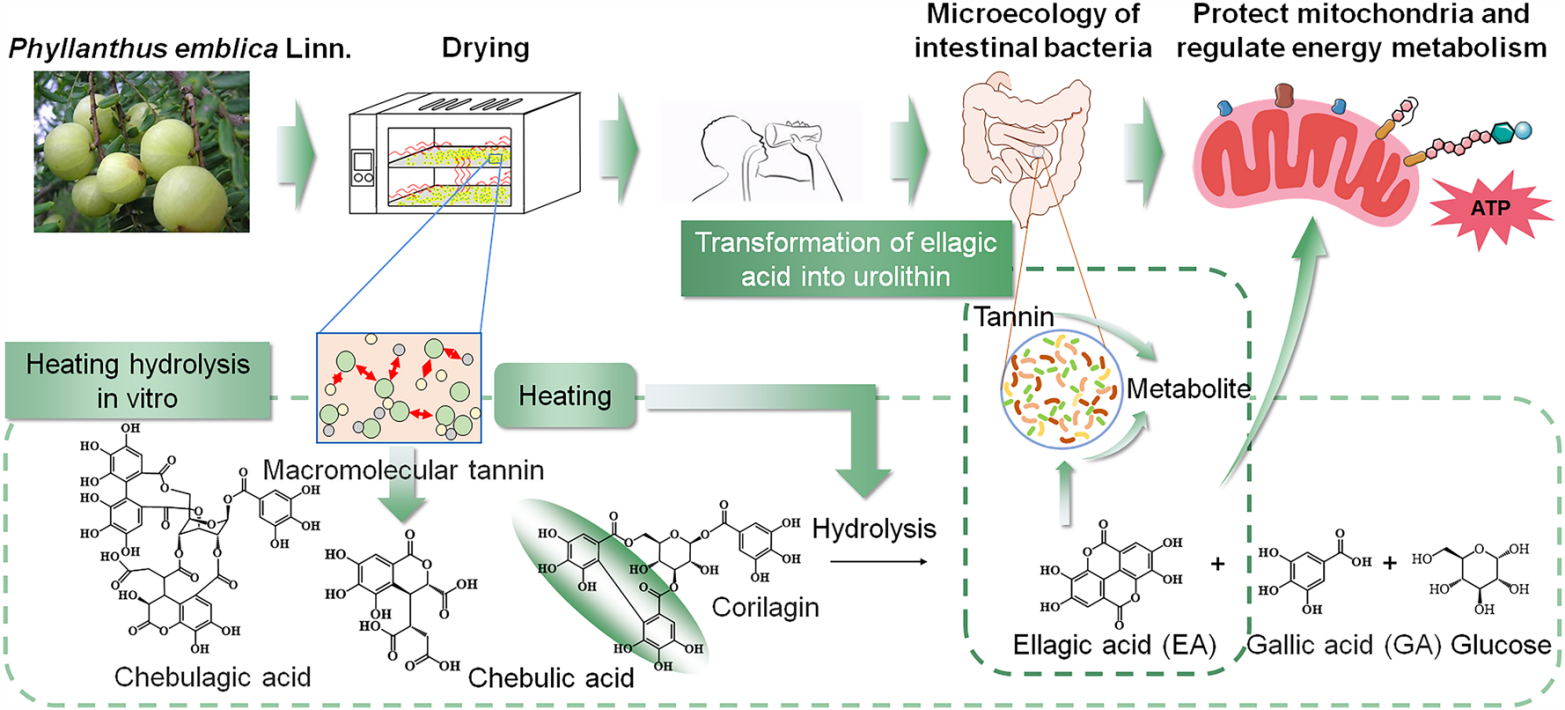
Schematic diagram of hydrolysis of tannin components in PE during processing

The anti-fatigue effect of PE will be impacted by the chemical change that occur throughout the drying processes. The anti-fatigue impact of PE changes dramatically after drying, and high temperature dried PE performs noticeably better than low temperature dried PE in terms of improving grip and exercise duration (treadmill, rotarod test, and loading swimming) as well as regulating the accumulation of harmful metabolites, oxidative stress, and energy balance in the body. This demonstrates that heat treatment will facilitate the exertion of PE’s anti-fatigue effect, during the processing of drying. It’s possible that the body is unable to absorb the PE’s macromolecular tannin. Under the influence of body enzymes, it must be digested into GA, EA, and other small molecule tannins. Then, intestinal flora transforms EA into additional metabolites such as protocatechuic acid derivatives and dibenzopyran-6-one metabolites, primarily urolithin [17]. However, because it takes time for this transformation to occur, some macromolecular tannins are discharged from the body before being hydrolyzed and absorbed in time, which results in the loss of a significant amount of anti-fatigue active components. The heat treatment during the drying encourages the hydrolysis of PE’s polyphenols and completes the conversion of PE’s macromolecular tannins to small molecular tannins in vitro in advance. This promotes PE’s absorption in the body, the exertion of its efficacy, and minimizes the loss of active ingredients, see Fig. 6. The correlation analysis result of PE also demonstrates that GA and EA, the final products of PE hydrolysis, are the primary constituents of PE for anti-fatigue.

Previous research has found that EA and GA have the capacity to inhibit astrocyte death and boost neuronal vitality, which may play an anti-fatigue role [32]. In addition, some metabolites of EA, such as urolitin, have also been shown to regulatory mitochondria through SIRT1-PGC-1α [9]. Now, this study found that EA and GA can increase the body's energy reserve, protect mitochondria, promote the synthesis of ATP, regulate the metabolism of sugars, lipids and amino acids, and affect the tricarboxylic acid cycle. All these indicate that the mechanism of EA and GA playing an anti-fatigue role is related to the protection of mitochondria and the regulation of energy metabolism. At the same time, EA and GA also have the ability to regulate intestinal flora, which mainly includes lactobacillus, bifidobacteriaceae, lactobacillus, clostridiales, eggerthellaceae, and Enterobacter, and EA’ s effect is stronger. Studies have found that some of these bacteria can alleviate fatigue by reducing the production of metabolites such as lactic acid, and the others are involved in the metabolism of EA. This shows that the regulation of intestinal flora by EA and GA is also one of the mechanisms of its anti-fatigue effect, through which the intestinal flora regulates fatigue related metabolites and promotes the transformation of the active components of PE in vivo.

In summary, heat treatment during processing is one of the methods to enhance the anti-fatigue effect of PE, which is related to the hydrolysis of PE polyphenols by heating to generate anti-fatigue active components EA and GA. And EA and GA can play an anti-fatigue role by protecting mitochondria, regulating energy metabolism and regulating gut flora, Meanwhile, EA has a stronger effect than GA, and EA is a key component of PE's resistance to exercise induced fatigue.

### Supplementary Information

**Additional file 1: Figure S1.** Correlation analysis of intestinal flora abundance (gene level). **S-2.** OPLS-DA score (A) and S-plot (B) of serum metabolites between PE group and model group under positive and negative ion mode. **S-3.** OPLS-DA score (A) and S-plot (B) of serum metabolites between EA group and model group under positive and negative ion mode. **S-4.** OPLS-DA score (A) and S-plot (B) of serum metabolites between GA group and model group under positive and negative ion mode. **S-5.** Differential metabolites in serum of PE and Model group under positive ion mode. **S-6.** Differential metabolites in serum of PE and Model group under negative ion mode. **S-7.** Differential metabolites in serum of EA and Model group under positive ion mode. **S-8.** Differential metabolites in serum of EA and Model group under negative ion mode. **S-9.** Differential metabolites in serum of GA and Model group under positive ion mode. **S-10.** Differential metabolites in serum of GA and Model group under negative ion mode.

## Data Availability

All data are fully available without restriction.
