# Supplementary material for: Thermal treatment enhances the resisting exercise fatigue effect of Phyllanthus emblica L.: novel evidence from tannin conversion in vitro, metabolomics, and gut microbiota community analysis
Source: Chin Med. 2023 Oct 1;18:127. doi: 10.1186/s13020-023-00835-4 (PMC10544184; doi:10.1186/s13020-023-00835-4)
Supplement: Supplementary file 1 — Additional file 1: Figure S1. Correlation analysis of intestinal flora abundance (gene level). S-2. OPLS-DA score (A) and S-plot (B) of serum metabolites between PE group and model group under positive and negative ion mode. S-3. OPLS-DA score (A) and S-plot (B) of serum metabolites between EA group and model group under positive and negative ion mode. S-4. OPLS-DA score (A) and S-plot (B) of serum metabolites between GA group and model group under positive and negative ion mode. S-5. Differential metabolites in serum of PE and Model group under positive ion mode. S-6. Differential metabolites in serum of PE and Model group under negative ion mode. S-7. Differential metabolites in serum of EA and Model group under positive ion mode. S-8. Differential metabolites in serum of EA and Model group under negative ion mode. S-9. Differential metabolites in serum of GA and Model group under positive ion mode. S-10. Differential metabolites in serum of GA and Model group under negative ion mode. [file 13020_2023_835_MOESM1_ESM.docx]

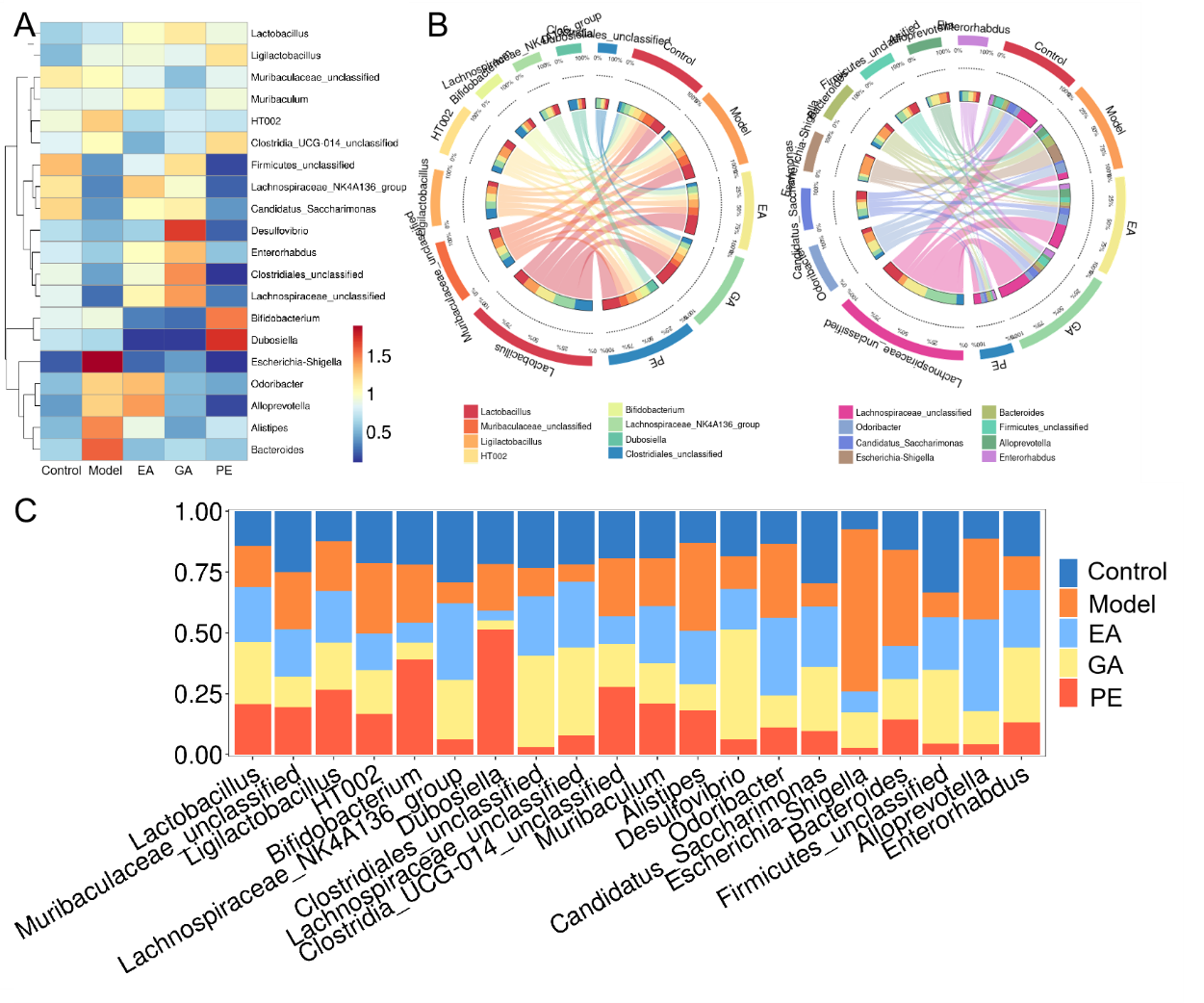


**S-1. Correlation analysis of intestinal flora abundance (gene level)**

**Note: A: Heat map; B: Circos (the left part is the abundance information of the gene with large abundance changes and its corresponding abundance information, and the right part is the grouping information); C: Histogram of intestinal flora abundance in each group**


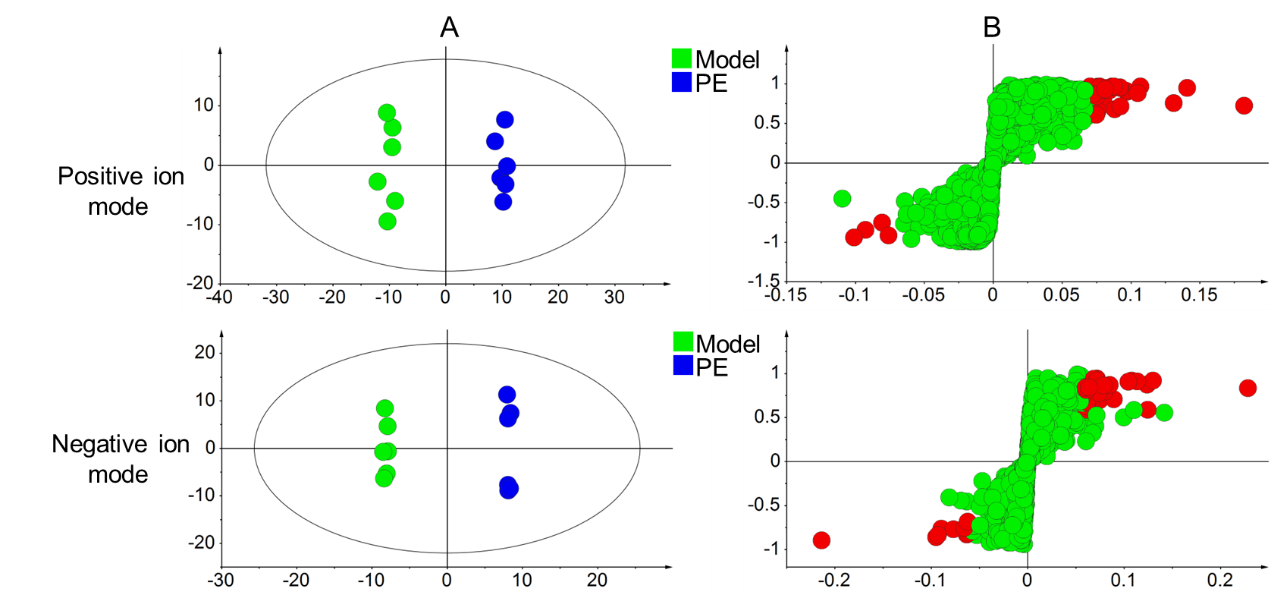


**S-2. OPLS-DA score (A) and S-plot (B) of serum metabolites between PE group and model group under positive and negative ion mode**


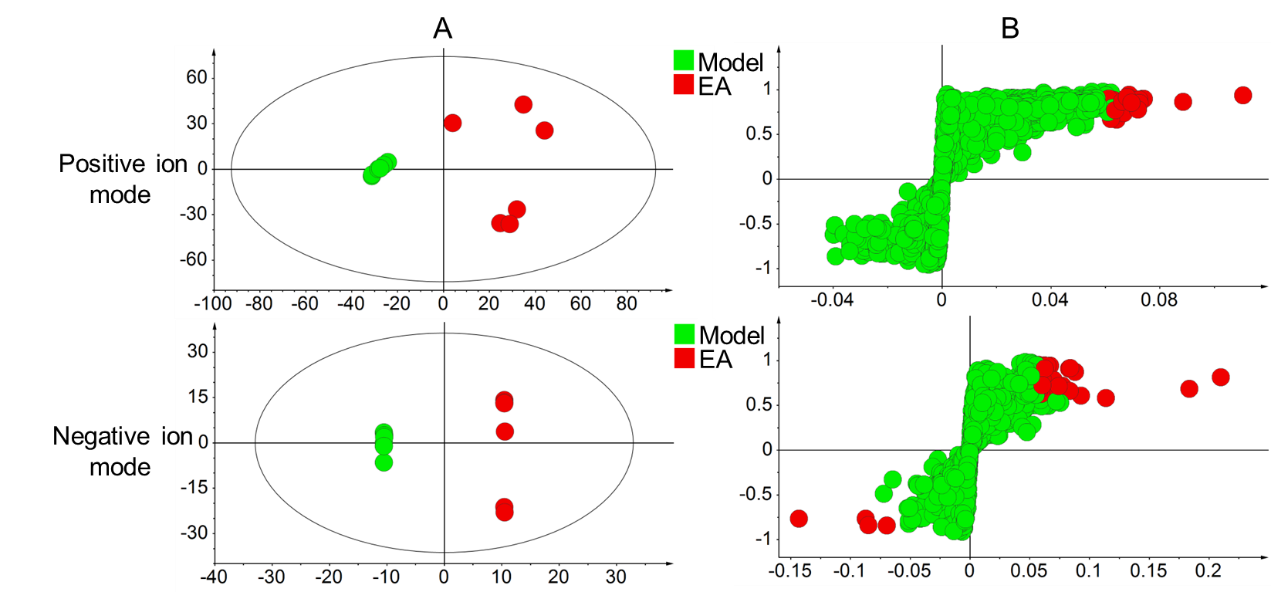


**S-3. OPLS-DA score (A) and S-plot (B) of serum metabolites between EA group and model group under positive and negative ion mode**


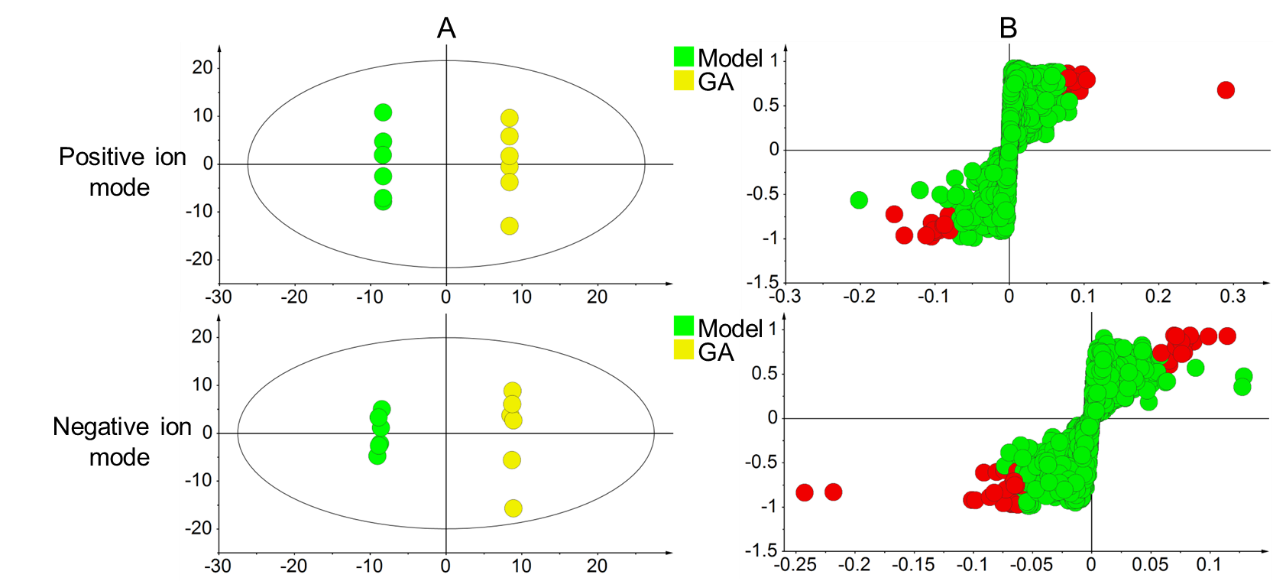


**S-4. OPLS-DA score (A) and S-plot (B) of serum metabolites between GA group and model group** **under positive and negative ion mode**

**S-5 Differential metabolites in serum of PE and Model group under positive ion mode**

| Component | Chemical formula | m/z | RT/min | Trend |
| --- | --- | --- | --- | --- |
| 2-Heptanone | C_7_H_14_O | 153.0663 | 2.00 | ↑ |
| Gibberellin A51 | C_19_H_24_O_5_ | 682.3621 | 17.17 | ↓ |
| Clupanodonyl carnitine | C_29_H_47_NO_4_ | 969.6889 | 20.56 | ↓ |
| Moreollin | C_35_H_42_O_8_ | 623.3257 | 21.18 | ↑ |
| SM(d18:0/20:0) | C_43_H_89_N_2_O_6_P | 799.6156 | 21.55 | ↑ |
| Ganolucidic acid A | C_30_H_44_O_6_ | 1064.6520 | 22.12 | ↑ |
| Adenosine tetraphosphate | C_10_H_17_N_5_O_16_P_4_ | 1196.9163 | 22.12 | ↑ |
| CDP-DG(i-24:0/i-24:0) | C_60_H_113_N_3_O_15_P_2_ | 1142.7469 | 23.69 | ↑ |
| 5,8-Epoxy-5,8-dihydro-10'-apo-b,y-carotene-3,10'-diol | C_27_H_38_O_3_ | 424.2973 | 24.63 | ↑ |
| 6-Glucopyranosylprocyanidin B2 | C_36_H_36_O_17_ | 758.2212 | 24.89 | ↑ |
| 5a-Cholestane-3a,7a,12a,25-tetrol | C_27_H_48_O_4_ | 911.6784 | 24.93 | ↑ |
| dCDP | C_9_H_15_N_3_O_10_P_2_ | 838.0664 | 25.02 | ↑ |
| 2-Hydroxyadipic acid | C_6_H_10_O_5_ | 201.0176 | 25.04 | ↑ |
| Octadecane | C_18_H_38_ | 550.6300 | 25.59 | ↓ |
| 6-Methylheptadecane | C_18_H_38_ | 550.6293 | 26.58 | ↑ |
| Acarbose | C_25_H_43_NO_18_ | 684.2038 | 26.61 | ↑ |
| Pelargonidin 3-sophoroside 5-glucoside | C_33_H_41_O_20_^+^ | 758.2190 | 27.12 | ↑ |

**S-6 Differential metabolites in serum of PE and Model group under negative ion mode**

| Component | Chemical formula | m/z | RT/min | Trend |
| --- | --- | --- | --- | --- |
| Toremifene | C_32_H_36_ClNO_8_ | 809.3657 | 13.88 | ↑ |
| 7-Hydroxy-6-methyl-8-ribityl lumazine | C_12_H_16_N_4_O_7_ | 327.0915 | 17.15 | ↑ |
| 3a,6b,7a,12a-Tetrahydroxy-5b-cholanoic acid | C_24_H_40_O_6_ | 469.2833 | 18.28 | ↑ |
| alpha-Irone | C_14_H_22_O | 457.3353 | 19.53 | ↑ |
| Behenoylglycine | C_24_H_47_NO_3_ | 378.3334 | 19.77 | ↑ |
| 3a,7a,12a-Trihydroxy-5b-cholestanoic acid | C_27_H_46_O_5_ | 449.3281 | 20.03 | ↑ |
| CDP-DG(18:2(9Z,11Z)/a-25:0) | C_55_H_99_N_3_O_15_P_2_ | 1138.6343 | 20.26 | ↑ |
| alpha-Chaconine | C_45_H_73_NO_14_ | 832.4833 | 20.31 | ↑ |
| Phenylephrine | C_9_H_13_NO_2_ | 500.3833 | 20.34 | ↑ |
| Citronellyl anthranilate | C_17_H_25_NO_2_ | 824.5510 | 20.51 | ↑ |
| Hexadecanedioic acid mono-L-carnitine ester | C_23_H_43_NO_6_ | 857.6080 | 21.55 | ↑ |
| PC(16:1(9Z)/22:2(13Z,16Z)) | C_46_H_86_NO_8_P | 856.6092 | 21.75 | ↑ |
| LysoPC(15:0/0:0) | C_23_H_48_NO_7_P | 480.4123 | 21.83 | ↑ |
| Biotin | C_10_H_16_N_2_O_3_S | 281.0358 | 22.76 | ↓ |
| N-((Hexahydro-1-azepinyl)carbonyl)-leucyl(1-methyl)-tryptophyl-tryptophan | C_34_H_45_N_5_O_6_ | 654.3126 | 22.85 | ↑ |
| PE(DiMe(11,5)/PGF2alpha) | C_47_H_82_NO_12_P | 928.5477 | 22.99 | ↓ |
| 3,28-Dihexadecanoyl 20(29)-lupene-2a,3b,28-triol | C_62_H_110_O_5_ | 969.8093 | 23.22 | ↑ |
| Josamycin | C_42_H_69_NO_15_ | 862.4423 | 24.49 | ↑ |
| N-Lauroyl Lysine | C_18_H_36_N_2_O_3_ | 701.5387 | 24.89 | ↑ |
| Forasartan | C_23_H_28_N_8_ | 397.2286 | 24.90 | ↓ |
| Cholesterol sulfate | C_27_H_46_O4S | 465.4043 | 27.15 | ↑ |
| Protocatechuic acid | C_7_H_6_O_4_ | 188.9943 | 27.19 | ↑ |

**S-7. Differential metabolites in serum of EA and Model group under positive ion mode**

| Component | Chemical formula | m/z | RT/min | Trend |
| --- | --- | --- | --- | --- |
| Camellioside D | C_54_H_88_O_24_ | 583.0235 | 10.55 | ↑ |
| D-Glucosaminide | C_18_H_35_N_3_O_13_ | 515.2337 | 11.72 | ↑ |
| PI(18:0/20:4(6Z,8E,10E,14Z)-2OH(5S,12R)) | C_47_H_83_O_15_P | 482.7636 | 14.88 | ↑ |
| CerP(d18:0/16:0) | C_34_H_70_NO_6_P | 620.5018 | 15.87 | ↑ |
| Sulfacytine | C_17_H_17_C_l2_N | 652.3433 | 16.13 | ↑ |
| (2R)-1-(4-Nonylphenyl)propan-2-amine | C_18_H_31_N | 523.4998 | 16.38 | ↑ |
| a-L-threo-4-Hex-4-enopyranuronosyl-D-galacturonic acid | C_24_H_32_O_24_ | 743.0925 | 16.45 | ↑ |
| cyclic GMP-AMP | C_20_H_24_N_10_O_13_P_2_ | 688.5869 | 16.75 | ↑ |
| 2-(3,4-Dihydroxyphenyl)-5,6-dihydroxy-7-methoxy-4H-1-benzopyran-4-one | C_16_H_12_O_7_ | 650.1546 | 17.19 | ↑ |
| Isorhamnetin 3-O-[b-D-glucopyranosyl-(1->2)-[a-L-rhamnopyranosyl-(1->6)]-b-D-glucopyranoside] | C_34_H_42_O_21_ | 828.4306 | 17.20 | ↑ |
| (S)-3-Hydroxytetradecanoyl-CoA | C_35_H_62_N_7_O_18_P_3_S | 994.1183 | 17.20 | ↑ |
| PC(O-16:0/22:0) | C_46_H_94_NO_7_P | 817.6804 | 17.82 | ↑ |

**S-8 Differential metabolites in serum of EA and Model group under negative ion mode**

| Component | Chemical formula | m/z | RT/min | Trend |
| --- | --- | --- | --- | --- |
| [(3R,4S)-1,1-Difluoro-3-(hexadecanoylamino)-4-hydroxy-4-phenylbutyl]phosphonic acid | C_26_H_44_F_2_NO_5_P | 554.2631 | 4.53 | ↑ |
| Vignatic acid B | C_27_H_41_N_3_O_7_ | 554.2634 | 5.04 | ↑ |
| alpha-(p-(1,1,3,3-Tetramethylbutyl)phenyl)-omega-hydroxypoly(oxyethylene) | C_14_H_22_O | 554.2633 | 9.54 | ↑ |
| Enkephalin L | C_28_H_37_N5O_7_ | 554.2648 | 10.54 | ↑ |
| Butirosina | C_21_H_41_N_5_O_12_ | 554.2623 | 11.04 | ↑ |
| Toremifene | C_26_H_28_ClNO | 809.3657 | 13.88 | ↑ |
| N-{(2R)-2-Hydroxy-2-[(8S,11S)-8-isopropyl-6,9-dioxo-2-oxa-7,10-diazabicyclo[11.2.2]heptadeca-1(15),13,16-trien-11-YL]ethyl}-N-isopentylbenzenesulfonamide | C_30_H_43_N_3_O_6_S | 554.2628 | 15.55 | ↑ |
| Phenylacetaldehyde | C_8_H_8_O | 285.1147 | 15.78 | ↑ |
| Tryptamine | C_10_H_12_N_2_ | 379.2143 | 16.56 | ↑ |
|  |  |  |  |  |
| Flavomycin | C_69_H_107_N_4_O_35_P | 790.3225 | 17.02 | ↑ |
| beta-Sinensal | C_15_H_22_O | 253.1362 | 17.57 | ↑ |
| L-Tryptophan | C_11_H_12_N_2_O_2_ | 467.1951 | 17.61 | ↑ |
| 13'-Hydroxy-gamma-tocotrienol | C_28_H_42_O_3_ | 471.3147 | 18.73 | ↑ |
| Glycyl-Tryptophan | C_13_H_15_N_3_O_3_ | 581.2419 | 18.75 | ↑ |
| Lubiprostone | C_20_H_32_F_2_O_5_ | 371.2081 | 18.87 | ↑ |
| 3a,7a,12a-Trihydroxy-5b-cholestanoic acid | C_27_H_46_O_5_ | 449.3281 | 20.03 | ↑ |
| Phenylephrine | C_9_H_13_NO_2_ | 500.3833 | 20.34 | ↑ |
| Orlistat | C_29_H_53_NO_5_ | 476.3799 | 20.36 | ↑ |
| 3-Hydroxyhexadecanoylcarnitine | C_23_H_45_NO_5_ | 452.3766 | 20.77 | ↑ |
| Squalamine | C_34_H_65_N_3_O_5_S | 664.4198 | 21.52 | ↓ |
| LysoPC(15:0/0:0) | C_23_H_48_NO_7_P | 480.4123 | 21.83 | ↑ |
| Dipivefrin | C_19_H_29_NO_5_ | 396.2008 | 22.91 | ↑ |
| Thalidomide | C_13_H_10_N_2_O_4_ | 747.6929 | 23.47 | ↑ |
| Artomunoxanthentrione | C_26_H_20_O_7_ | 887.2329 | 27.17 | ↑ |
| Citrusin F | C_22_H_32_O_14_ | 259.5852 | 27.18 | ↑ |

**S-9. Differential metabolites in serum of GA and Model group under positive ion mode**

| Component | Chemical formula | m/z | RT/min | Trend |
| --- | --- | --- | --- | --- |
| Cotinine methonium ion | C_11_H_15_N_2_O^+^ | 174.1148 | 5.51 | ↓ |
| Porson | C_22_H_26_O_6_ | 428.2095 | 11.18 | ↑ |
| Histidinyl-Isoleucine | C_12_H_20_N_4_O_3_ | 559.2913 | 13.84 | ↓ |
| Phenazopyridine | C_11_H_11_N_5_ | 468.2393 | 16.77 | ↑ |
| D-Maltose | C_12_H_22_O_11_ | 343.1215 | 18.82 | ↑ |
| 5-Hydroxyflavone | C_21_H_20_O_8_ | 433.1517 | 19.03 | ↑ |
| 4a-Hydroxytetrahydrobiopterin | C_9_H_15_N_5_O_4_ | 578.2422 | 19.41 | ↓ |
| PC(16:0/22:6(5Z,7Z,10Z,13Z,16Z,19Z)-OH(4)) | C_46_H_80_NO_9_P | 411.7838 | 19.66 | ↑ |
| Mycophenolic acid | C_17_H_20_O_6_ | 641.2663 | 19.69 | ↓ |
| DG(16:0/20:4(5Z,8Z,11Z,14Z)/0:0) | C_39_H_68_O_5_ | 661.4802 | 21.56 | ↑ |
| Hericenone H | C_37_H_54_O_6_ | 1189.7949 | 21.58 | ↑ |
| 3-O-beta-D-glucosyl-brassicasterol | C_34_H_56_O_6_ | 1159.7907 | 21.91 | ↑ |
| Arginyl-Isoleucine | C_12_H_25_N_5_O_3_ | 638.4069 | 21.99 | ↑ |
| Ganglioside GA2 (d18:1/24:0) | C_62_H_116_N_2_O_18_ | 1159.8232 | 22.17 | ↑ |
| 5-Hydroxycapsanthin | C_39_H_54_O_4_ | 1173.8027 | 22.18 | ↑ |
| Isofucosterol glucoside | C_35_H_58_O_6_ | 1187.8206 | 22.18 | ↑ |
| 16a-Hydroxydehydroisoandrosterone | C_19_H_28_O_3_ | 672.4246 | 22.19 | ↑ |

**S-10. Differential metabolites in serum of GA and Model group under negative ion mode**

| Component | Chemical formula | m/z | RT/min | Trend |
| --- | --- | --- | --- | --- |
| Vignatic acid B | C_27_H_41_N_3_O_7_ | 554.2634 | 5.04 | ↓ |
| LysoPC(18:3(6Z,9Z,12Z)/0:0) | C_26_H_48_NO_7_P | 554.2638 | 6.04 | ↓ |
| L-Aspartic acid | C_4_H_7_NO_4_ | 168.0071 | 6.27 | ↓ |
| Gentisate aldehyde | C_7_H_6_O_3_ | 183.0311 | 6.27 | ↓ |
| LysoPC(18:3(9Z,12Z,15Z)/0:0) | C_26_H_48_NO_7_P | 554.2637 | 9.04 | ↓ |
| alpha-(p-(1,1,3,3-Tetramethylbutyl)phenyl)-omega-hydroxypoly(oxyethylene) | C_14_H_22_O | 554.2633 | 9.54 | ↓ |
| Butirosina | C_21_H_41_N_5_O_12_ | 554.2639 | 12.04 | ↓ |
| Tragopogonsaponin K | C_50_H_72_O_15_ | 949.4407 | 13.85 | ↑ |
| Toremifene | C_32_H_36_ClNO_8_ | 809.3657 | 13.88 | ↓ |
| 2-[4,6-Bis(2,4-dimethylphenyl)-1,3,5-triazin-2-yl]-5-(octyloxy)phenol | C_33_H_39_N_3_O_2_ | 530.2837 | 15.90 | ↑ |
| 2-Arachidonylglycerol | C_23_H_38_O_4_ | 423.2767 | 17.24 | ↑ |
| Spironolactone | C_24_H_32_O_4_S | 415.1995 | 17.25 | ↓ |
| Citronellyl formate | C_11_H_20_O_2_ | 183.1395 | 17.78 | ↓ |
| Glycyl-Tryptophan | C_13_H_15_N_3_O_3_ | 581.2419 | 18.75 | ↓ |
| PE(DiMe(13,5)/MonoMe(13,5)) | C_52_H_92_NO_10_P | 942.6162 | 18.78 | ↑ |
| PI(16:0/18:0) | C_43_H_83_O_13_P | 837.5570 | 18.79 | ↑ |
| Malvidin 3-rutinoside | C_29_H_35_O_16_^+^ | 660.1714 | 18.81 | ↓ |
| 10,11-dihydro-20-trihydroxy-leukotriene B4 | C_20_H_34_O_7_ | 385.2245 | 19.48 | ↓ |
| Phenylephrine | C_9_H_13_NO_2_ | 500.3833 | 20.34 | ↓ |
| Pregnanediol | C_21_H_36_O_2_ | 365.2731 | 21.36 | ↑ |
| Squalamine | C_34_H_65_N_3_O_5_S | 664.4198 | 21.52 | ↑ |
| LysoPC(15:0/0:0) | C_23_H_48_NO_7_P | 480.4123 | 21.83 | ↓ |
